# Disentangling the relationships among abundance, invasiveness and invasibility in trait space

**DOI:** 10.1038/s44185-023-00019-1

**Published:** 2023-06-09

**Authors:** Cang Hui, Petr Pyšek, David M. Richardson

**Affiliations:** 1 Centre for Invasion Biology, Department of Mathematical Sciences, Stellenbosch University, Stellenbosch, South Africa; 2 Biodiversity Informatics Unit, African Institute for Mathematical Sciences, Muizenberg, South Africa; 3 National Institute for Theoretical and Computational Sciences (NITheCS), Stellenbosch University, Stellenbosch, South Africa; 4 Institute of Botany, Czech Academy of Sciences, Prague, Czech Republic; 5 Department of Ecology, Charles University, Prague, Czech Republic; 6 Centre for Invasion Biology, Department of Botany & Zoology, Stellenbosch University, Stellenbosch, South Africa

**Keywords:** Community ecology, Invasive species, Theoretical ecology

## Abstract

Identifying conditions and traits that allow an introduced species to grow and spread, from being initially rare to becoming abundant (defined as invasiveness), is the crux of invasion ecology. Invasiveness and abundance are related but not the same, and we need to differentiate these concepts. Predicting both species abundance and invasiveness and their relationship in an invaded community is highly contextual, being contingent on the community trait profile and its invasibility. We operationalised a three-pronged invasion framework that considers traits, environmental context, and propagule pressure. Specifically, we measure the invasiveness of an alien species by combining three components (performance reflecting environmental suitability, product of species richness and the covariance between interaction strength and species abundance, and community-level interaction pressure); the expected population growth rate of alien species simply reflects the total effect of propagule pressure and the product of their population size and invasiveness. The invasibility of a community reflects the size of opportunity niches (the integral of positive invasiveness in the trait space) under the given abiotic conditions of the environment. Both species abundance and the surface of invasiveness over the trait space can be dynamic and variable. Whether an introduced species with functional traits similar to those of an abundant species in the community exhibits high or low invasiveness depends largely on the kernel functions of performance and interaction strength with respect to traits and environmental conditions. Knowledge of the covariance between interaction strength and species abundance and these kernel functions, thus, holds the key to accurate prediction of invasion dynamics.

## The mystery of species abundance in communities

Individuals are the fundamental units of species. The number of individuals distributed over space and time thus provides a direct measure of the numerical performance of species^1^. Consequently, the abundance of a target species can be seen as a barometer for comparing its demographic performance against those of co-occurring species. The ability to estimate and predict the level and trend of abundance for target species at a spatial scale relevant to management, especially considering the unprecedented global and regional environmental changes that are currently underway, sets the goal for modern population ecology^2^.

Species abundance can be measured along three intrinsically correlated dimensions:^3,4^ local population density, geographic range, and niche breadth. Specifically, geographic range and population density are related via a probability rule known as the occupancy-abundance relationship^5^, while widespread species also typically possess large niche breadths^6^. All three dimensions strongly affect population viability and are indicative of the ability of a species to become invasive if moved out of its native range. Although species with different levels of abundance arguably possess different traits and are influenced by different assembly processes^7,8^, it is still challenging to predict the expected level of abundance of a species based only on its demographic and functional traits, despite the many theories and explanations that have been proposed to explain species abundance and rarity.

At any given time, most species in an assemblage are rare, and only a few are abundant; the species-abundance distribution at fine spatial scales is almost universally skewed and shows a J-shaped lognormal or similar form^9,10^. Diverse schools of thought and approaches have yielded clues about the level of evenness among species in a community, although a robust foundation for explaining and especially predicting species abundance remains elusive^10^. For instance, metabolic theory sets a maximum density for a species of a given size to pack its individuals into available resource landscapes^11^; this is known as the size-density relationship^12–14^ although it only “paint[s] nature with a very broad brush”^15^. Nevertheless, a theory-based predictive framework is a laudable aim as it would facilitate true comprehension and extrapolation on how species, communities and habitats respond to global change drivers and how introduced and resident species perform in mixed and highly transformed ecosystems. This is the aim of the present work.

Through human-mediated dispersal and biological invasions, the exchange of individuals between locations is accelerating, not only for alien species but also for resident (native) ones^16–18^. Alien and native species are expected to respond to different assembly processes, as do rare and abundant species^19^. How biological invasions affect resident species of different abundances and how fast an introduced species becomes abundant or gets expelled from a recipient ecosystem require clarification. We set out to develop a theory-based predictive model to tentatively address these demands. This model allows us (i) to predict the invasiveness of an introduced species and the invasibility of the recipient community based on their relative positions in the trait space and (ii) assess the relationship between species abundance and invasiveness in open transformative communities under persisting invasions. We first pull together threads from invasion science that provide the foundation for this theoretical framework and then introduce the framework using mathematical notations. With this model, we highlight the peculiar role played by rare or newly introduced alien species and explain the inflation or deflation of invasiveness by the covariance between the interaction strength and abundance of residing species in the recipient community. A glossary of essential concepts and terminology used appears in Table 1.

**Table 1:** A glossary of key concepts and terms of the three-pronged framework and model.

Term	Interpretation
Abundance	The numerical performance of a species in a community; often reported as population size or density. It is the most basic dimension of the commonness and rarity of a species.
Community resilience	The capacity of a community to maintain its structure, functioning and feedbacks despite shocks and perturbations. Here, shocks refer particularly to the impacts of biological invasions on the demographic performance of resident species.
Essential biodiversity variables (EBVs)	A set of basic biodiversity indicators that provide information on population viability and ecosystem resilience.
Invasibility	The properties of a community that determine its inherent vulnerability to invasion. In this work, it is the integral of trait regions with positive invasiveness.
Invasiveness	The features of an alien organism, such as its life-history traits, that define its capacity to invade (establish and spread) following introduction through human agency. The level of invasiveness of a species can change over time and can be measured by the invasion growth rate, defined as the rate of intrinsic growth when the number of alien propagules is trivial while the abundances of resident species fluctuate around their equilibria.
Limiting similarity	A concept in theoretical ecology and community ecology that proposes the existence of a maximum level of niche overlap between two given species that will allow continued coexistence.
Opportunity niche	The view that ecological communities with specialised interactions could hamper the effect of radiation and coevolution, resulting in empty niches unexploited from incremental evolution. The presence of such empty niches in communities thus create opportunities for alien species to establish and exploit through ecological fitting (the emergence and formation of biotic interaction without the coevolution of involved species, but through matching of compatible traits, often after rapid trails and learning).
Propagule pressure	A concept that encompasses variation in the quantity, quality, composition, and rate of supply of alien organisms, or their propagules, resulting from the transport conditions and pathways between source and recipient regions.
Species coexistence	Multiple mechanisms mediate species coexistence and invasion performance in a community. For species coexistence through mutual invasibility, fitness equivalence (or reduced fitness difference) is necessary, while for biological invasions fitness advantage is emphasised over fitness equivalence. In the two-species model, fitness difference is measured by the difference of their intrinsic rates of growth, while stabilising force such as niche overlap, or the lack of niche segregation, is measured by comparing interspecific and intraspecific interaction strengths^67^.

## A three-pronged invasion framework

Invasion dynamics are context-dependent and non-equilibrial^20,21^. The invasiveness of an alien species reflects its demographic performance, while multiple ecological and evolutionary processes can, directly or indirectly, regulate invasiveness via a complex network of causal pathways^22^. The invasiveness of an alien species in a community can be measured by its expected initial per-capita population growth rate, also known as the invasion growth rate^23^. The invasibility of the recipient ecosystem, on the other hand, depends on the community trait profile (i.e. how residing species are located relative to each other in the trait space) and reflects the size of opportunity niches (i.e., trait space with positive invasiveness)^24^. Invasibility is, therefore, a measure of community openness (often signalled by dynamic instability and temporal compositional turnover), while invasiveness is the capacity to occupy any existing opportunity niches with preadapted traits via ecological fitting^25^ or the ability to create such opportunity niches by hampering community resilience and even destabilising the recipient ecosystem^26,27^.

**Fig. 1:**
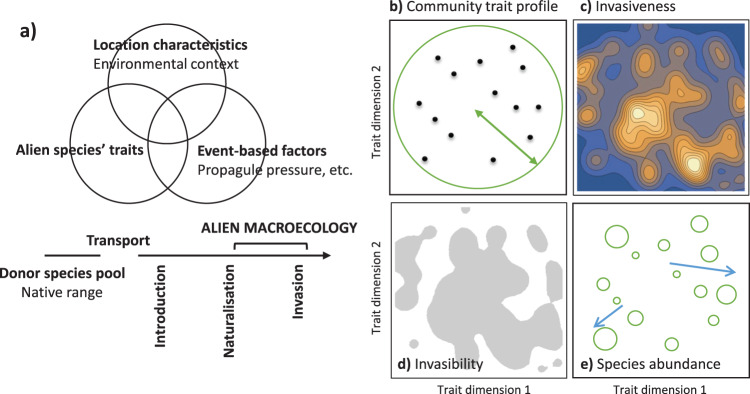
Key concepts and patterns of the three-pronged invasion framework and associated model in trait space. **a** A typology of factors, represented by intersections in the Venn diagram that explain invasions and differentiated along the introduction-naturalisation-invasion continuum, where alien macroecology refers to the richness, distribution, abundance, spatial and trait relationships of alien biota at large spatial scales^32^. **b** A community trait profile, represented by the trait positions of resident species (black dots) in the two-dimensional trait space. The double-headed arrow within the green circle indicates the trait centroid and the trait periphery of the community trait profile. **c** The surface of invasiveness, calculated as the invasion growth rate (see Table 1 for explanation) for any given trait position. Blue to white colours indicate invasion growth rate from negative to positive values. **d** Invasibility of the invaded community, represented by the size of grey areas that experience positive invasiveness. **e** Each resident species fluctuates around a particular abundance, indicated by the size of a green circle. Abundance gradients among these resident species, represented by blue arrows, can be identified locally in the trait space. Abundance gradients do not necessarily conform to the gradient of invasiveness (**c**) or the centrality of trait position (**b**); rather, all three jointly emerge in the open community transition and turnover as a result of persistent biological invasions.

Many destabilising mechanisms have been proposed as contending invasion hypotheses, and their complexity has propelled invasion science to edge forward following a simplified three-pronged approach (Fig. 1a), highlighting the roles of propagule pressure, invasive traits, and environmental context^28–32^. First, there is the umbrella effect of propagule pressure that strongly influences the establishment success of an alien species^33,34^ and its performance over later invasion stages^35^. Propagule pressure reflects the associated introduction pathways^36,37^, as well as the taxon’s physiological tolerance during transport^38,39^. Second, certain life-history traits are associated with invasion success^40,41^, although this is highly taxon-specific^42^. For instance, invasive plants possess traits associated with high fecundity, efficient dispersal capacity, and the ability to utilise generalist mutualists and evade specific natural enemies^40,43^. Finally, identified invasive traits are context-dependent and often have poor transferability for prediction^44^. This is because invasion outcomes and impacts within a community are entangled by interactions between the invader’s traits and the invaded ecosystem^45^. One needs to consider the ecological similarity between the native and non-native ranges in terms of habitat, resource, disturbance, and co-occurring species, all of which regulate the performance of an invader and moderate the opportunity niche that can be realised by the invader in its new home.

Over the last twenty years, invasion science has experienced a flourish of frameworks, hypotheses and models to describe or explain highly unpredictable invasion outcomes in order to coordinate management efforts to mitigate invasion impacts^46,47^. In an attempt to advance the theoretical framework of invasion science from the linear form of introduction-naturalisation-invasion continuum^48,49^ and to synthesise the consensus of the three-pronged approach, Pyšek et al.^32^ proposed a macroecological framework for biological invasions (MAFIA) by invoking these three clusters of factors and their interactions to capture the contextual dependence of invasion performance: alien species’ traits (e.g., fast/slow strategy, body size, niche breadth, fecundity, native range size); site characteristics (e.g., temperature range, resource availability, native community, disturbance); and event-related factors (e.g., colonisation and propagule pressure, residence time, season, pathway) (Fig. 1a). The three-pronged method was projected over the introduction-naturalisation-invasion continuum, to highlight different factors at work along the continuum The predictability of invasion success and performance can arguably be improved when all three prongs of factors are considered in combination. We operationalise this MAFIA framework by projecting the three-pronged framework in the trait space and highlight the difference and relationship among key features emerging through this framework. These include the centrality in the community trait profile (Fig. 1b), invasiveness as measured by the invasion growth rate for any candidate alien species (Fig. 1c), invasibility as indicated by the areas of empty niches in the trait space (Fig. 1d), and species relative abundances in a community (Fig. 1e).

## Defining invasiveness and invasibility

Let *n* be the population size of an alien species. According to the three-pronged framework, the anticipated population change rate $\dot{n}$ of this alien species can be written as a function of propagule pressure (*γ*, the influx rate of alien propagules), its traits *x* (a vector of multiple measured traits), and the abiotic and biotic environment of the recipient ecosystem (*E* and *S*, respectively, with the biotic component specified as the abundance ${n}_{i}$ and traits ${x}_{i}$ of the $S$ residing species). A minimum equation that explicitly describes the expected rate of population change of the alien species as the combination of these three-pronged factors is^50^:

1 $$\dot{n}=\gamma +r\left(x\,|\,E\right)n+\sum _{i\in S}\alpha (x,{x}_{i}{\rm{|}}E){n}_{i}n$$

where $r\left(x\,|\,E\right)$ is the functional performance (fitness) of the alien species (specified by its traits) under the abiotic environmental condition of the recipient site, which represents the environmental suitability of the site; $\alpha (x,{x}_{i}{|E})$ depicts the per-capita biotic interaction strength (positive or negative pressure) of resident species to the alien species in the abiotic environment of the recipient ecosystem, with ${n}_{i}n$ proportional to the encounter rate between individuals of two species.

For simplicity, we only consider these demographic rates ($r$ and $\alpha$) dependent on traits and the environment. This model is only a generic description of the three-pronged framework as the exact kernel functions of performance ($r$) and interaction strength ($\alpha$) with respect to traits ($x$ and ${x}_{i}$) and the environment ($E$) are unspecified. These rates can also depend on current or past densities, for instance, when considering lag phase and Allee effect (for $r$) and nonlinear functional response (for $\alpha$). However, a few key invasion features are already rendered explicit through this model. The umbrella effect of propagule pressure becomes apparent as the anticipated initial population growth rate of the alien species reflects solely the influx rate of alien propagules ($\dot{n}=\gamma$ when $n=0$). Note, propagule pressure does not necessarily lead to alien establishment if the environment is unsuitable ($r\left(x\,|\,E\right) \,<\, 0$); this is evident when the influx of alien propagules halts. After removing this umbrella effect of propagule pressure, we see that the invasiveness ($f$) of an introduced species depends on its traits and the environment^24^: $f({x|E},S)=r\left(x\,|\,E\right)+\sum _{i\in S}\alpha (x,{x}_{i}{|E}){n}_{i}$, which can be assessed before the introduction. To elucidate the factors in this definition, let ${\alpha }_{S}={\{\alpha (x,{x}_{i}{|E})\}}_{i\in S}$ represent the vector of per-capita biotic interaction strength from the resident species to the alien species, and ${n}_{S}={\{{n}_{i}\}}_{i\in S}$ the abundance vector of all resident species. We can define the invasiveness in the following mathematically equivalent way (Fig. 1c):

2 $$f\left(x\,|\,E,S\right)=r\left(x\,|\,E\right)+S\,\cdot\, {\rm{cov}}\left({\alpha }_{S},{n}_{S}\right)+{\bar{\alpha }}_{S}N$$

where $N$ is the community size (total number of individuals in the recipient ecosystem) and ${\bar{\alpha }}_{S}$ the average per-capita biotic interaction strength imposed by resident species. Evidently, invasiveness reflects the combination of three components: (i) an invader’s performance in the abiotic environment ($r\left(x\,|\,E\right)$), (ii) total biotic interaction pressure from the entire recipient ecosystem (${\bar{\alpha }}_{S}N$), and (iii) the product of resident species richness ($S$) and the covariance between biotic interaction strength (${\alpha }_{S}$) and resident species abundance (${n}_{S}$). The expected invasion dynamics is simply the total of propagule pressure and the product of the alien population size and its invasiveness, $\dot{n}=\gamma +n\,\cdot\, f\left(x\,|\,E,S\right)$.

The invasibility ($g$) of the recipient ecosystem can be calculated as the integral of the opportunity niches (i.e., trait positions with positive invasiveness)^24^ (Fig. 1d):

3 $$g\left(E,S\right)=\int {\rm{IF}}(f\left(x{\rm{|}}E,S\right) \,>\, 0{\rm{;}}1,0){dx}$$

where IF(logic; true, false) is the simple conditional function. Consequently, invasibility examines all possible invasion outcomes for any given invasive traits. Invasiveness is a function of an invader’s traits conditioned upon the context of the recipient ecosystem, whereas invasibility is an emergent property of the ecosystem’s abiotic and biotic environment. If an alien species possesses the traits within an opportunity niche of a community, it can establish and invade. Importantly, the opportunity niche and invasibility can be dynamic due to community assembly (${n}_{S}[t]$, $S[t]$), biological invasions ($n[t]$), and environmental fluctuations ($E(t)$)^51,52^. The above definitions of invasion dynamics ($\dot{n}$), invasiveness ($f\left(x\,|\,E,S\right)$), and invasibility ($g\left(E,S\right)$) are rooted in the three-pronged invasion framework. In the next section we highlight some research undercurrents that can make these abstract definitions practical and measurable.

## Invasiveness and abundance in trait space

There is an abundance gradient among resident species in the trait space for any ecological community, a staircase of commonness and rarity (Fig. 1e). We need to understand not only the mechanisms that create this gradient but also the drivers of the waxing and waning of species abundance on this staircase. While a native or an alien species can climb this staircase to become common (and invasive in the case of an alien species), an abundant species may step down the same staircase to become rare and even extinct^53^. Of course, rarity does not necessarily imply extinction. For instance, endangered species are certainly rare, but not all rare species are close to extinction^54^. So, what is the gatekeeper of this rarity staircase? If the pre-invasion community is closed (no immigration) and resilient to small perturbations, the abundance vector of resident species will settle around the equilibrium ${\hat{n}}_{S}=-{r}_{S}{A}_{S}^{-{\rm{T}}}$, with ${r}_{S}={\{r\left({x}_{i}\,|\,E\right)\}}_{i\in S}$ and ${A}_{S}{=\{\alpha ({x}_{i},{x}_{j}{|E})\}}_{i,j\in S}$, representing the vector of performance and the matrix of the interaction strength between resident species. Near this equilibrium, the community dynamics can be approximated by a linear dynamical system with the Jacobian matrix, ${\rm{diag}}({\hat{n}}_{S}){A}_{S}$, while the community resilience requires the real part of the lead eigenvalue of this matrix to be negative (${\rm{Re}}\left({\lambda }_{L}\right) \,<\, 0$).

If we consider biological invasions as perturbations to a community, successful invasions will drive compositional turnover and community transition^27^, implying that the pre-invasion equilibrium is unstable (${\rm{Re}}\left({\lambda }_{L}\right) \,>\, 0$). Although instability and invasibility are not equivalent to each other, invasibility ($g\left(E,S\right)$) can be indicated nonetheless by the magnitude of the lead eigenvalue (${\lambda }_{L}$, a measure of community instability)^24^, with the anticipated community turnover proportional to the associated eigenvector^27^. This is not passing the challenge for quantifying invasibility on to another abstract measure, as theoretical studies suggest that the lead eigenvalue can be approximated as ${\lambda }_{L}\approx \alpha ({x}_{1},{x}_{1}{|E}){\hat{n}}_{1}$, where ${\hat{n}}_{1}$ is the abundance of the rarest species and $\alpha ({x}_{1},{x}_{1}{|E})$ [typically negative] its self-regulation coefficient^55,56^. Due to such peculiar roles of rare species in determining community resilience and signalling invasibility, an influx of rare alien propagules can easily flip a community from marginal stability to instability, initiating and propelling community transition^57^. This counter-intuitive role of rare species also corroborates their important functional roles^58^, as perturbations to the abundance and viability of rare species can have the most profound effect on the system stability^59–62^. For instance, losing rare species could lead to a greater reduction of functional specialisation, species richness, and community originality than would be the case with random species loss^63^. Indeed, recent studies have confirmed that species with small population sizes are responsible for the persistent temporal turnover in ecological networks^64^. Understanding how rare species are regulated in a community thus explains how an introduced species moves along the rarity staircase and fares in a recipient ecosystem.

How are invasiveness and species abundance distributed in the community trait space (Fig. 1c, e)? Are they aligned with the core-peripheral structure of the community trait profile (Fig. 1b)? In other words, are resident species occurring in the centre of the community trait profile more abundant? Are alien species located at the periphery of the community trait profile more invasive? In her classic paper, besides proposing the seven forms of rarity, Rabinowitz^3^ also contemplated the causal relationship between a species’ population size and its competitiveness (akin to invasiveness). She concluded that competitiveness is not a regulator of population size but mainly a strategy to offset the disadvantage of being locally rare. In other words, invasiveness is not necessarily related to abundance but reflects the ability of an alien species to overcome its initial rarity (i.e., the invasion growth rate^23,65,66^).

We can clarify this point further by elucidating the different forces at play in our model using a simple two-species scenario (species 1 native and species 0 alien). Consider the scenario that the alien species was introduced accidentally by a one-off event and initially had a trivial population size while the population size of the native species is set at its equilibrium (${\hat{n}}_{1}=-r({x}_{1}{|E})/\alpha ({x}_{1},{x}_{1}{|E})$), the relative growth ratio of the invader to the native species is, therefore, ${\dot{n}}_{0}/{n}_{0}-{\dot{n}}_{1}/{n}_{1}=\left(r({x}_{0}{|E})-r({x}_{1}{|E})\right)+{\hat{n}}_{1}\left(\alpha \left({x}_{0},{x}_{1}{|E}\right)-\alpha ({x}_{1},{x}_{1}{|E})\right)$. The right-hand side of this ratio includes two parts:^67^ fitness difference ($r({x}_{0}{|E})-r({x}_{1}{|E})$) and stabilising force (with a complete niche overlap if $\alpha \left({x}_{0},{x}_{1}{|E}\right)=\alpha ({x}_{1},{x}_{1}{|E})$ and complete niche segregation if $\alpha \left({x}_{0},{x}_{1}{|E}\right)=0$); see Table 1 for the explanation of specialised terms. The invader can only succeed if it has a higher fitness ($r\left({x}_{0}\,|\,E\right) \,>\, r({x}_{1}{|E})$), or greater niche segregation ($\alpha \left({x}_{0},{x}_{1}{|E}\right) \,>\, \alpha ({x}_{1},{x}_{1}{|E})$). Niche segregation from abundant native species is especially necessary for invasion success if there is no fitness advantage or difference between the alien and the resident species (i.e., a neutral case).

**Fig. 2:**
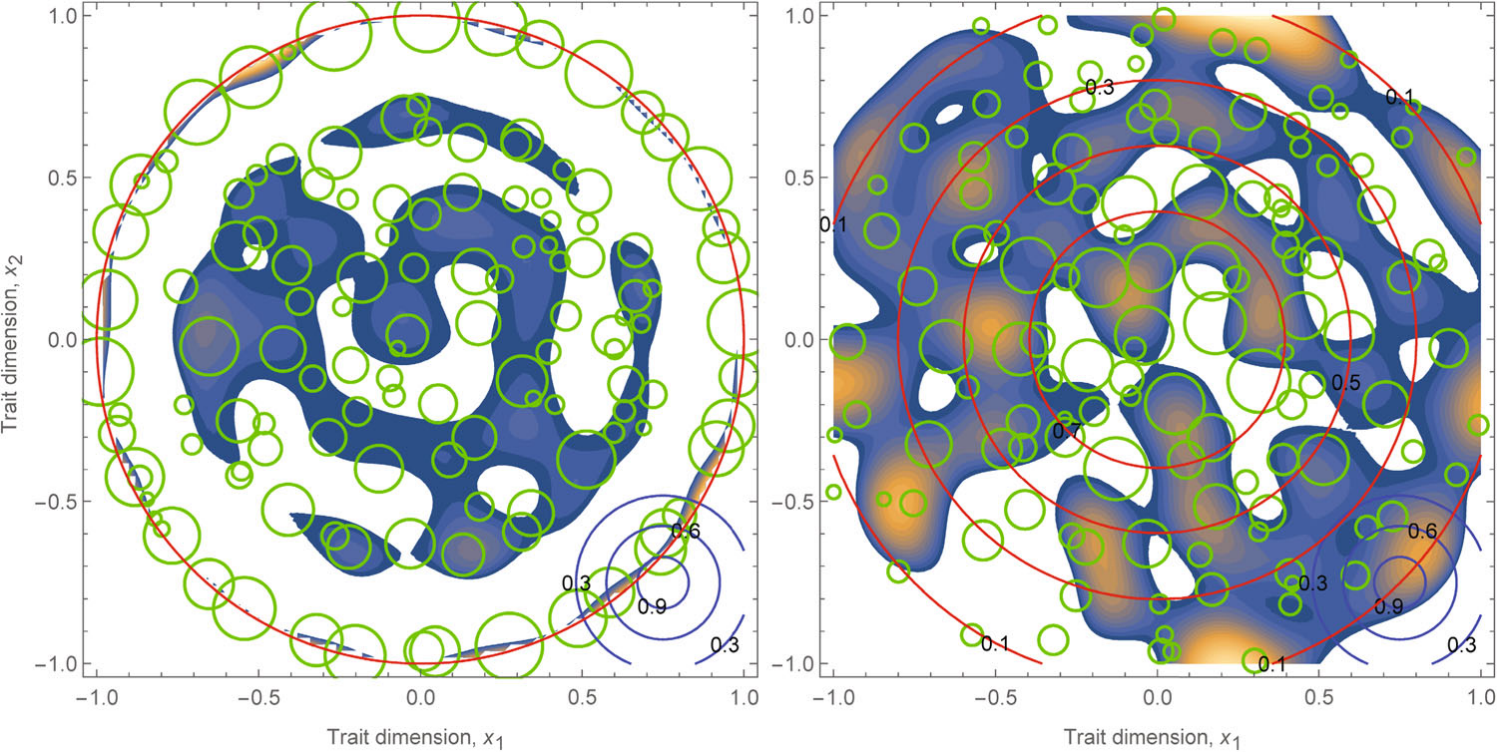
Illustrations of invasiveness, invasibility and species abundance in the trait space of open communities affected by persistent biological invasions. For visualisation, the trait space is presented as a two-dimensional plane (${x}_{1}$ and ${x}_{2}$); in practice, trait space is hyperdimensional where each dimension is a measured trait, while a lower dimensional visualisation such as this can be achieved using ordination. Trait values can be considered as rescaled values with respect to the centroid of the trait profile. Open community assembly and invasion processes were implemented according to the model (see Supplementary Information); specifically, we sequentially introduce species with randomly assigned trait positions and a small initial propagule size ($n\left[t=0\right]=0.01$) into the community according to a Poisson process at a rate of 1.2 introduction events per unit of time. Plots reflect snapshots of communities at $t=1000$ when the community is fluctuating around a stationary state. The centre and size of green circles represent the trait position and abundance of a species at the time of observation. The blue-orange surface represents the level of positive invasiveness (opportunity niches), with the areal size indicating invasibility and the colour indicating invasiveness at a particular trait position. An invader possessing traits within the blue-orange surface establishes at the time of observation, while those outside the surface fail. The demonstration requires us to specify the kernel functions of performance and interaction strength in the three-pronged model, and we provide two cases with specified kernel functions. Left: $r\left({x|E}\right)=0.5$ within the unit circle and zero outside the unit circle; Right: $r\left({x|E}\right)={{\exp }}(-{d}_{x,0}^{2}/0.7)-0.1$; both: $\alpha \left(x,y\,|\,E\right)={{\exp }}(-{d}_{x,y}^{2}/0.06)$, where ${d}_{x,y}$ is the Euclidean distance of positions $x$ and $y$ in the two-dimensional trait space. To visualise these two kernel functions, we drew red circles for the contours of performance kernel $r$ and blue circles in the bottom right for the contours of interaction strength kernel $\alpha$. See detailed model implementation^50^ and code in the Supplementary Information. Importantly, how tightly species can be packed into the trait space reflects the limiting similarity for species coexistence (Table 1), while the formation of self-organised opportunity niches (trait positions with positive invasiveness) and species abundances (Left) reflects the phenomena of emergent neutrality^75^ and hidden niches^76^. The abundance gradient and invasiveness can be correlated with each other (Left) or not (Right), and they can be either aligned with the trait centroid-periphery structure (Right) or not (Left).

Once the specific forms of the kernel functions of these demographic rates ($r$ and $\alpha$) are known, the actual community transition as a result of persistent biological invasions can be simulated using our model. For instance, assuming that interaction strength between species 1 and 2 becomes stronger when the two species has more similar traits $\alpha \left({x}_{1},{x}_{2}\,|\,E\right)={\rm{exp }}(-{d}_{\mathrm{1,2}}^{2}/{\sigma }_{\alpha }^{2})$ and that the intrinsic rate of growth is trait independent $r\left({x|E}\right)={c}_{1}$ or declines from the centroid to the periphery in the trait space $r\left({x}_{1}{|E}\right)={\rm{exp }}(-{d}_{\mathrm{1,0}}^{2}/{\sigma }_{r}^{2})-{c}_{2}$, where ${d}_{\mathrm{1,2}}$ is the Euclidean distance of positions ${x}_{1}$ and ${x}_{2}$ in the two-dimensional trait space and others are model parameters dependent on traits and the environment (see Supplementary Information), the patterns outlined in the three-pronged framework (Fig. 1) emerged dynamically in the trait space driven by persistent invasions (Fig. 2). It is evident that the distribution of and the relationship between invasiveness and species abundance in the trait space, as illustrated in Fig. 2, depends on the forms of kernel functions of performance and interaction strength ($r$ and $\alpha$) with respect to traits and environmental conditions ($x$ and $E$). Consequently, given the trait profile of a multispecies community, trait positions of alien species that impose a more positive interaction strength-abundance covariance (${\rm{cov}}\left({\alpha }_{S},{n}_{S}\right)$) will optimise niche differentiation and augment the invasiveness^50,68^; this optimal niche differentiation for elevated invasion performance is typically, although not exclusively, found at the edge of the trait space^69,70^. Indeed, when comparing invasive species with the entire trait profile of the invaded community (i.e., the cloud of resident species in the trait space), invasive species possess more distinct traits compared to native and naturalised species in many real communities^41,69^. In ecological networks with multiple functional guilds and interaction types, more complex patterns can emerge; for instance, elevated invasive performance can be found in the trait space in such complex ecological networks that ensures higher fitness gain from more abundant mutualists or lower fitness loss from more abundant competitors or antagonists^50^.

The applicability of this model relies on progress in two fronts. First, the kernel functions that relate demographic rates and interaction strengths to relative trait positions (and values) need to be supported by empirical data. Second, functional traits of all species in the invaded community, or at least of those within the interaction network of a focal invasive species, must be known. The first one is a key endeavour of the research field that implements the trait paradigm of community ecology and network ecology^57,71,72^. More tests are needed to ensure that the forms and relationships of these kernels are strong enough for downstream applications, although applications of different kernel functions have been theoretically explored in evolutionary ecology^50,73^. The second front has seen a few studies in invasion ecology that document anticipated transitions in community trait profiles due to biological invasions^69^. Empirical tests using real data on propagule pressure, traits and abundances of all residing species of an ecological community are still lacking. This is because, besides these model parameters, estimates of community transition caused by biological invasion for testing the predictability of this model often require repeated surveys of the invaded communities over long periods, which are only starting to be attempted.

## Conclusions

To assess invasion performance according to the three-pronged framework of traits, environmental context, and propagule pressure^32^, we can define and measure invasiveness by combining three components: a performance that reflects fitness and environmental suitability; product of community species richness and the covariance between interaction strength and species abundance; and community-level interaction pressure. The expected population growth rate of an alien species simply reflects the combined effect of propagule pressure and the product of population size and invasiveness. Invasibility can thus be measured as the size of opportunity niches (positive invasiveness in the trait space) given the abiotic conditions of the environment. In practice, invasibility reflects the loss of community resilience and can be signalled by compositional temporal turnover, especially the gain and loss of rare species. The gradient of rarity and the distribution of invasiveness in the trait space can be dynamic and variable; both are largely dependent on the kernel functions of performance and interaction strength ($r$ and $\alpha$) with respect to traits and environmental conditions ($x$ and $E$). Consequently, invasion monitoring should not only strive to quantify all essential biodiversity variables^74^, but also the interaction strength-abundance covariance (${\rm{cov}}\left({\alpha }_{S},{n}_{S}\right)$) that inflates or hampers of performance alien species invasion, as well as the unspecified kernel functions of performance and interaction strength ($r$ and $\alpha$) with respect to traits and environmental conditions ($x$ and $E$). The knowledge of this covariance and these unspecified kernel functions holds the key to accurate prediction of invasion dynamics and is flagged as a research priority.

### Supplementary information

Supplementary Information

## Data Availability

This article does not contain data.

## Code availability

The computer code for producing Fig. 2 and supporting the conclusions is available in the Supplementary Information. The code is written in Mathematica 12.0 (Wolfram, Inc.) and can be accessed without restrictions.
