# Supplementary material for: Disentangling the relationships among abundance, invasiveness and invasibility in trait space
Source: NPJ Biodivers. 2023 Jun 9;2:13. doi: 10.1038/s44185-023-00019-1 (PMC11332024; doi:10.1038/s44185-023-00019-1)
Supplement: Supplementary file 1 — Supplementary Information [file 44185_2023_19_MOESM1_ESM.pdf]

Supplementary Information: Mathematica code to produce Fig.2.
Part of, Hui, C., Pyšek, P. & Richardson, D.M. (2022) Disentangling the relationships among abundance, invasiveness and invasibility in trait space. npj Biodiversity

The code is prepared by C. Hui using Mathematica 12.0 (02/11/2022; Wolfram, Inc.). The code running requires about 5 min to complete on a Dell OptiPlex 7060 with Intel(R) Core(TM) i7-8700 CPU and 16 GB RAM. Below, we provide a brief introduction to the code structure.

The model implements an open ecological community under persistent once-off biological invasions. All residing species within the community, also including the introduced alien species, engage in resource competition with an interaction strength determined by the traits of the two invovled species. The intrinsic rate of growth of each species is also trait dependent. In a nutshell, a species' trait position in the community trait profile deterimines its performance and interactions with other species. For jargons and expanded code, please see Table 1 and main text, as well as Hui et al. 2021 (https://doi.org/10.1007/s10530-021-02484-w).

First, we define the kernel functions of r (intrinsic rate of growth) and a (interaction strength [competition] between two species) in the two dimensional trait space (x, y). Coefficients siga and sigr are related to the specific shapes of the kernel functions. Inner functions of Mathematica are intutitve; chop[] removes small positive numbers close to zeros, Exp[] is the exponential function, If[] is for conditional, and Sqrt[] is for square root calucaltion; see Mathematica Help for more details.These two parameterised kernel functions are for the left plot in Fig.2.

```
siga = 0.03; sigr = 0.35;
a[x1_, y1_, x2_, y2_] := Chop[Exp[-((x1 - x2)^2 + (y1 - y2)^2) / (2 siga)]];
r[x1_, y1_] := If[Sqrt[((x1)^2 + (y1)^2)] < 1, 0.5, 0];
```

Second, the introduction events of alien species are pre-defined using a Poisson process (with u the intensity [number of introduction events per unit of time]) to specify the timing of each event, with the trait of the introduced species (x, y) randomly drawn in the trait space (-1<x<1, -1<y<1). A total of 10,000 introduction events are prepared and will be sequentially introduced into the community at the time according to vector *tt*. Please see Mathematica Help for inner functions (PoissonProcess[], RandomFunction[], Dimensions[], and RandomReal[]).

```
u = 1.2;
tt = RandomFunction[PoissonProcess[u], {0, 10000}]["Path"][[All, 1]];
tn = Dimensions[tt][[1]];
x = RandomReal[{-1, 1}, tn];
y = RandomReal[{-1, 1}, tn];
```

Third, the following is the main part of the code. It specifies the ordinary differential equations (ODEs) for survived residents and newly introduced: *comm* describes the generic Lotka-Volterra model with trait-mediated performance and interactions (r and a), where *set* is the dynamic species identity set, *len* the number of species in the community, and *abu* the abundance of each species in the community. The ODEs are then solved by NDSolve[] between two introduction events and the solution assigned to *sol*. All of these are written within a loop (Do[] for *k* introduction events; for

demonstration, we consider a number of *k* = *tmax* introduction events here but can be modified if needed. A species is considered extinct if its standing population density *n* is less than a threshold (0.01; can be modified). Note, *n* records population densities for all species, whereas *abu* only for those currently in the community.

```
Clear[t]
set = {1}; len[1] = Length[set]; spp[0] = set; abu[0] = {0.01};
comm = Flatten[
   Table[{n[i]'[t] == n[i][t] (r[x[[i]], y[[i]]] - Sum[a[x[[i]], y[[i]], x[[j]],
              y[[j]]] × n[j][t], {j, set}]), n[i][tt[[1]]] == 0.01}, {i, set}]];
sol = NDSolve[comm, Table[n[i], {i, set}], {t, tt[[1]], tt[[2]]}];
spp[1] = set;
abu[1] = Evaluate[Table[n[i][tt[[2]]], {i, set}] /. sol][[1]];
tmax = 1000;
Monitor[Do[{
   ext = Table[If[n[i][tt[[k]]] < 0.01, True, False], {i, set}] /. sol[[1]];
   pos = Flatten[Position[ext, True], 1];
   set = Union[Complement[set, set[[pos]]], {k}];
   len[k] = Length[set];
   comm = Flatten[Table[{n[i]'[t] == n[i][t] (r[x[[i]], y[[i]]] -
            Sum[a[x[[i]], y[[i]], x[[j]], y[[j]]] × n[j][t], {j, set}]),
        n[i][tt[[k]]] == If[i == k, 0.01, n[i][tt[[k]]] /. sol[[1]]]}, {i, set}]];
   sol = NDSolve[comm, Table[n[i], {i, set}], {t, tt[[k]], tt[[k + 1]]},
     Method → {"EquationSimplification" → "Residual"}];
   spp[k] = set;
   abu[k] = Evaluate[Table[n[i][tt[[k + 1]]], {i, set}] /. sol][[1]]
  }, {k, 2, tmax}], k]
```

Additional metrics can be computed by the following code, which records each species time of entry, time of extirpation, duration, and abundance dynamics during its residence time in the community:

```
Do[{ time=Flatten[Position[Table[Length[Cases[spp[i],k]],{i,tmax}],1]];
tbegin[k]=Min[tt[[time]]]; tend[k]=Max[tt[[time]]]; life[k]=tend[k]-tbegin[k];
ab=Flatten[Table[abu[t][[Flatten[Position[spp[t],k]]]],{t,time[[1]],time[[-1]]}]];
abm[k]=Mean[ab]; abme[k]=Median[ab]; abma[k]=Max[ab]; abp[k]=ab;
},{k,1,tmax}]
```

Finally, the following code is for visualisation and will generate the left plot of Fig.2. Specifically, it overlays a number of plots. It visualizes population densities/abundances as green circles, invasiveness as contour surface, kernel function of r as red contour circles, and competition kernel of a as blue contour circles. Please see the legend of Fig.2 for details.

In[ ]:=
```
t = tmax;
fig1 = Graphics[Table[{Green, Thickness[0.0045], Circle[{x[[i]], y[[i]]},
      0.2 Sqrt[abu[t][[Position[spp[t], i][[1]]]][[1]]]]}, {i, spp[t]}],
   Frame → True, PlotRange → {{-1, 1}, {-1, 1}}];
fig2 = ContourPlot[a[0.75, -0.75, x1, y1], {x1, -1, 1}, {y1, -1, 1},
   ContourStyle → Blue, Contours → {0.3, 0.6, 0.9},
   PlotRange → All, ContourLabels → True, ContourShading → None];
fig3 = Graphics[{Red, Circle[{0, 0}, 1]}];
cen =
  Table[r[xref, yref] - Sum[a[xref, yref, x[[spp[t][[j]]]], y[[spp[t][[j]]]]] ×
      abu[t][[j]], {j, len[t]}], {xref, -1, 1, 0.02}, {yref, -1, 1, 0.02}];
tx = ListContourPlot[cen, DataRange → {{-1, 1}, {-1, 1}}, Contours → 20,
   PlotRange → {0, All}, ContourStyle → None];
Show[tx, fig1, fig2, fig3, FrameLabel → {"Trait dimension, x1 ",
   "Trait dimension, x2 "}, PlotRange → {{-1, 1}, {-1, 1}}]
```

Note, a graphic will be produced from the code above (Fig.2 left).

The following code will produce the right plot in Fig.2. The only difference between the left and the right plot is the kernel function of r. Above, r is a constant within the unit disk; below, r is a Gaussian function centred at the origin of the trait space minus a constant (to sink the intrinsic growth rate surface so that it becomes negative outside the unit disk). The first three steps of the above code are simply lumped together below into one block.

In[ ]:=
```
r[x1_, y1_] := Max[0, Exp[- ((x1) ^2 + (y1) ^2) / (2 sigr)] - 0.1];
(*for Fig.1 Right*)
(*Open community assembly via sequential species introductions*)
Clear[t]
set = {1}; len[1] = Length[set]; spp[0] = set; abu[0] = {0.01};
comm = Flatten[
   Table[{n[i]'[t] == n[i][t] (r[x[[i]], y[[i]]] - Sum[a[x[[i]], y[[i]], x[[j]],
            y[[j]]] × n[j][t], {j, set}]), n[i][tt[[1]]] == 0.01}, {i, set}]];
sol = NDSolve[comm, Table[n[i], {i, set}], {t, tt[[1]], tt[[2]]}];
spp[1] = set;
abu[1] = Evaluate[Table[n[i][tt[[2]]], {i, set}] /. sol][[1]];
tmax = 1000;
Monitor[Do[{
   ext = Table[If[n[i][tt[[k]]] < 0.01, True, False], {i, set}] /. sol[[1]];
   pos = Flatten[Position[ext, True], 1];
   set = Union[Complement[set, set[[pos]]], {k}];
   len[k] = Length[set];
   comm = Flatten[Table[{n[i]'[t] == n[i][t] (r[x[[i]], y[[i]]] -
            Sum[a[x[[i]], y[[i]], x[[j]], y[[j]]] × n[j][t], {j, set}]),
        n[i][tt[[k]]] == If[i == k, 0.01, n[i][tt[[k]]] /. sol[[1]]]}, {i, set}]];
   sol = NDSolve[comm, Table[n[i], {i, set}], {t, tt[[k]], tt[[k + 1]]},
     Method → {"EquationSimplification" → "Residual"}];
   spp[k] = set;
   abu[k] = Evaluate[Table[n[i][tt[[k + 1]]], {i, set}] /. sol][[1]]
  }, {k, 2, tmax}], k]
```

The code below is for visualisation of the right plot of Fig.2; specifics are the same as above and detailed in the figure legend.

In[•]:=

```
t = tmax;
fig1 = Graphics[Table[{Green, Thickness[0.0045], Circle[{x[[i]], y[[i]]},
      0.2 Sqrt[abu[t][[Position[spp[t], i][[1]]]][[1]]]]}, {i, spp[t]}],
   Frame → True, PlotRange → {{-1, 1}, {-1, 1}}];
fig2 = ContourPlot[a[0.75, -0.75, x1, y1], {x1, -1, 1}, {y1, -1, 1},
   ContourStyle → Blue, Contours → {0.3, 0.6, 0.9},
   PlotRange → All, ContourLabels → True, ContourShading → None];
fig3 = ContourPlot[r[x1, y1], {x1, -1, 1}, {y1, -1, 1},
   Contours → {0.1, 0.3, 0.5, 0.7, 0.9}, ContourStyle → Red,
   PlotRange → All, ContourLabels → True, ContourShading → None];
cen = Table[r[xref, yref] - Sum[a[xref, yref, x[[spp[t][[j]]]], y[[spp[t][[j]]]]] ×
      abu[t][[j]], {j, len[t]}], {xref, -1, 1, 0.02}, {yref, -1, 1, 0.02}];
tx = ListContourPlot[cen, DataRange → {{-1, 1}, {-1, 1}}, Contours → 20,
   PlotRange → {0, All}, ContourStyle → None];
Show[tx, fig1, fig2, fig3, FrameLabel → {"Trait dimension, x1 ",
   "Trait dimension, x2 "}, PlotRange → {{-1, 1}, {-1, 1}}]
```

--- The End ---
